# Cost effectiveness of therapeutic drug monitoring for imatinib administration in chronic myeloid leukemia

**DOI:** 10.1371/journal.pone.0226552

**Published:** 2019-12-23

**Authors:** Kibum Kim, Gwendolyn A. McMillin, Philip S. Bernard, Srinivas Tantravahi, Brandon S. Walker, Robert L. Schmidt

**Affiliations:** 1 Department of Pathology and ARUP Laboratories, University of Utah, Salt Lake City, Utah, United States of America; 2 Department of Pharmacy, Pharmacotherapy Outcomes Research Center, University of Utah, Salt Lake City, Utah, United States of America; 3 Department of Internal Medicine, Division of Hematology and Hematological Malignancies, University of Utah, Salt Lake City, Utah, United States of America; The University of Adelaide, AUSTRALIA

## Abstract

**Background:**

Imatinib mesylate (IM) is a first-line treatment option for patients with chronic myeloid leukemia (CML). Patients who fail or are intolerant to IM therapy are treated with more expensive second and third-generation tyrosine kinase inhibitors. Patients show wide variation in trough concentrations in response to standard dosing. Thus, many patients receive subtherapeutic or supratherapeutic doses. Therapeutic drug monitoring (TDM) may improve dose management that, in turn, may reduce costs and improve outcomes. However, TDM also adds to the cost of patient care. The objective of this study was to determine the cost-effectiveness of TDM for generic IM therapy.

**Methods:**

We developed a microsimulation model for the trough plasma concentration of IM which is related to a cytogenetic or molecular response. We compared two cohorts: one with TDM and one without TDM (NTDM). The lifetime incremental cost-effectiveness ratio (ICER) was calculated using quality-adjusted life years (QALYs) as the effectiveness measure. One-way and probabilistic sensitivity analyses were performed.

**Results:**

The lifetime cost and QALY of treatment with TDM were $2,137K [95% Ci: 2,079K; 2,174K] and 12.37 [95% CI: 12.07; 12.55], respectively. The cost and QALY of NTDM were $2,132K [95% CI: 2,091K; 2,197K] and 12.23 [95% CI: 11.96; 12.50], respectively. The incremental cost and QALY for TDM relative to NTDM was $4,417 [95% CI: -52,582; 32,097]) and 0.15 [95% CI: -0.13; 0.28]. The ICER for TDM relative to NTDM was $30,450/QALY. Probabilistic sensitivity analysis showed that TDM was cost-effective relative to NTDM in 90% of the tested scenarios at a willingness-to-pay threshold of $100,000/QALY.

**Conclusions:**

Although the impact of TDM is modest, the cost-effectiveness over a lifetime horizon (societal perspective, ($30,450/QALY) falls within the acceptable range (< $100k/QALY).

## Introduction

Chronic myeloid leukemia (CML) is a myeloproliferative neoplasm that accounts for approximately 15% of the incidence of adult leukemia.[1] One out of every 500 to 600 individuals is diagnosed with CML in his or her lifetime.[1, 2] In almost all cases, CML is caused by a BCR-ABL gene translocation (i.e., Philadelphia chromosome positive). Life expectancy for CML patients has significantly improved since the advent of tyrosine kinase inhibitors (TKIs) to the extent that survival is largely driven by non-CML related mortality. As a result, the prevalence of CML has increased substantially.[3] CML treatment is costly and can exceed $100,000 per year. This, combined with the increased prevalence of CML, has led to high individual and societal costs of CML treatment.[4, 5]

Imatinib mesylate (IM) was the first TKI approved for the treatment of CML. Since then, several other TKIs have been introduced (e.g., dasatinib, nilotinib, bosutinib and ponatinib) in order to overcome the resistance mediated by kinase domain inhibitors. Second generation TKIs (2GTKI), dasatinib and nilotinib, are approved for front line therapy based on two separate randomized studies.[6, 7] Bosutinib, another 2GTKI was initially approved for second-line agent and more recently received FDA approval for newly diagnosed CML.[8] Ponatinib, a third-generation TKI (3GTKI), is available for patients after failure of at least two TKIs or in the presence of a T315I mutation.[9] The choice of front line TKI, IM versus 2GTKI, is often decided based on comorbidities and expected toxicity profile. An additional factor to consider in determination of choice of front line TKI is cost of treatment. The newer TKIs are called second-generation TKIs (2GTKIs). More recently, third-generation TKIs (e.g., ponatinib) have been introduced. IM lost patent protection in 2016 and, as a result, the price began to decrease. Depending on the cost, IM could become the most cost-effective alternative for treatment of CML.[10, 11] Given this scenario, IM would be preferred to 2GTKIs for front line therapy and other TKI options would be reserved for the relapse/refractory setting or intolerance.[10, 12] Savings would be proportional to the time that patients remain on generic IM before switching to an expensive 2GTKI. Thus, there is an incentive to find ways to minimize the failure rate of IM therapy to minimize costs and improve outcomes.

Failure occurs most commonly due to emergence of IM resistant BCR-ABL1 kinase domain mutations.[13] Patients who adhere to the scheduled regimen are more likely to response to IM treatment.[14] Moreover, the dose of IM is standard for all patients; however, some require dose reduction for toxicity or dose escalation due to lack of response. In these situations, therapeutic drug monitoring (TDM) can provide an individual patient’s IM pharmacokinetic (PK) profile and help physicians make informed dosing decisions.

Patients show wide variation in trough concentrations in response to standard dosing.[15] Thus it is likely that some patients receive subtherapeutic doses whereas others supratherapeutic doses. Several recent reviews have suggested that long-term oral cancer therapy fills many of the criteria for effective TDM.[15–17] These include the following: absence of an easily measurable biomarker for drug effect, long term therapy, availability of an analytical method, significant variability in pharmacokinetic exposure, narrow therapeutic range, defined and consistent expose-response relationships, and feasible dose-adaptation strategies. Imatinib fulfills many of these criteria.

Unfortunately, there is little evidence on the efficacy of TDM for adjusting IM dosing. Most studies on TDM have been observational and designed to determine relationships between trough concentration and response. To our knowledge, there has only been one prospective trial in which the incremental benefits of TDM (in addition to molecular monitoring) have been evaluated.[18] This study compared “routine” TDM (dose adjustment based on TDM for all patients) vs “rescue TDM” (dose adjustment based on treatment failure or toxicity). An intention to treat analysis found that TDM provided no benefit; however, this was likely due to the size of the study (N = 23 in each arm) and the fact that there was very poor adherence with respect to dose adjustment recommendations in the intervention arm (routine TDM). An “as treated” analysis found that routine TDM provided a statistically significant benefit among patients who were treated according to the dose adjustment protocol. This suggests that TDM can be effective if prescribers adhere to the protocol. However, it is not known whether TDM is cost-effective. The objective of this study was to explore the conditions under which TDM of IM levels could be cost effective in the context of CML treatment.

## Methods

### Model overview

The objective of this model-based study is to determine the cost effectiveness of therapeutic drug monitoring (TDM) for IM therapy administered for CML. A microsimulation model compared two treatment arms: one with TDM (TDM arm) and one without TDM (NTDM arm). As per current practice, that both cohorts would continue to receive molecular monitoring every three months.[19] Thus, in the TDM arm, patients were monitored by TDM and molecular monitoring. In the NTDM arm, patients were followed only by molecular monitoring. The primary outcomes are cost and Quality Adjusted Life Years (QALYs). Costs and protocols were based on practice in the United States.

**Fig 1:**
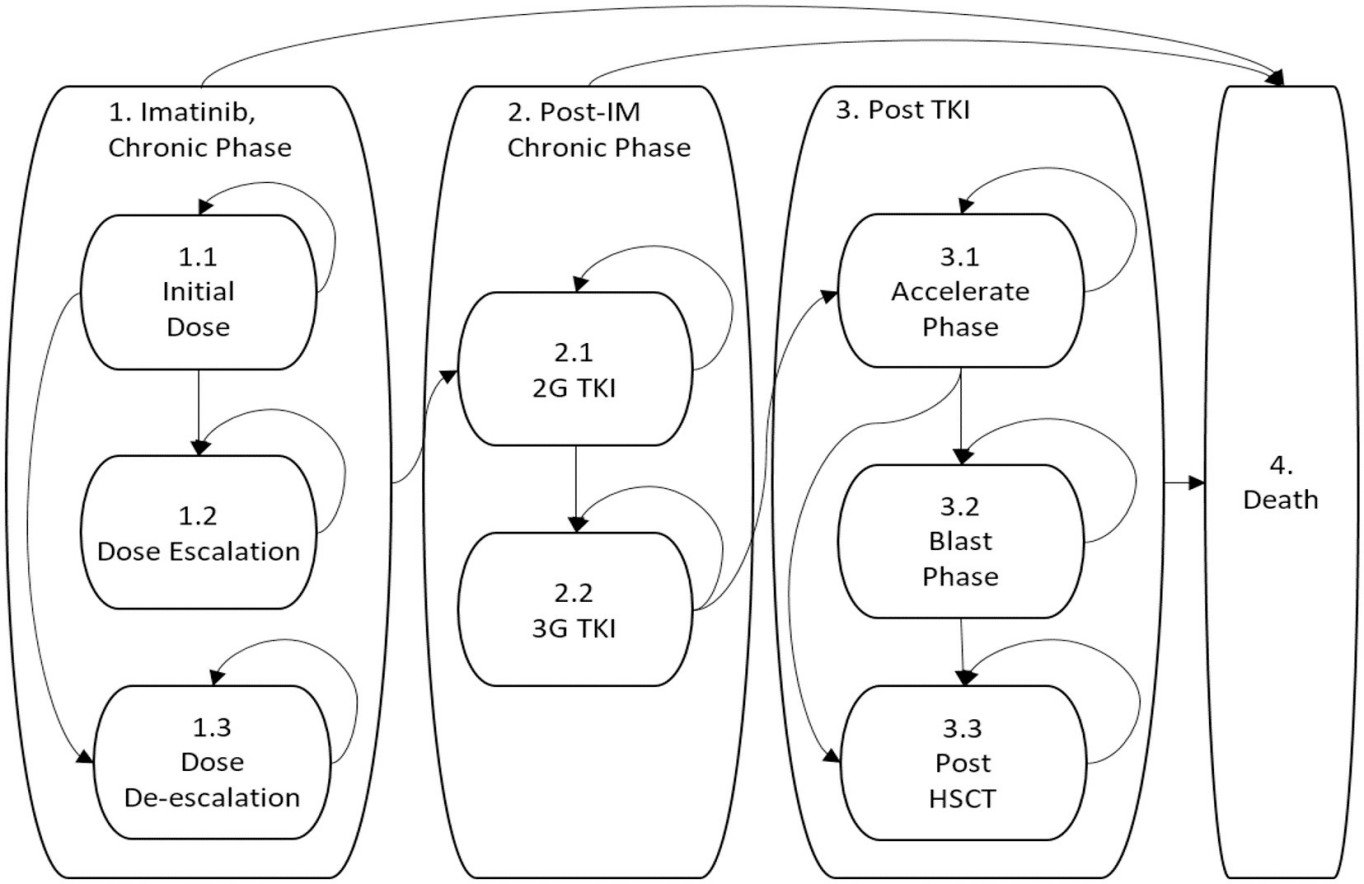
State-transition diagram. Patients with chronic myeloid leukemia begin enter the model immediately after diagnosis in the untreated state. They are initially given imatinib (state 1.1) and are subject to either dose escalation or de-escalation depending on response and/or intolerance. If imatinib therapy is unsuccessful, patients are given a second-generation tyrosine kinase inhibitor (2GTKI) and followed by a third-generation tyrosine kinase inhibitor (3GTKI, ponatinib). Patients remain on the 2GTKI or 3GTKI as long as the treatment is successful. Patients move to post-TKI therapy if all the TKI fails. Treatment fails if the patient becomes intolerant or is resistant. TKI = Tyrosine Kinase Inhibitor.

**Fig 2:**
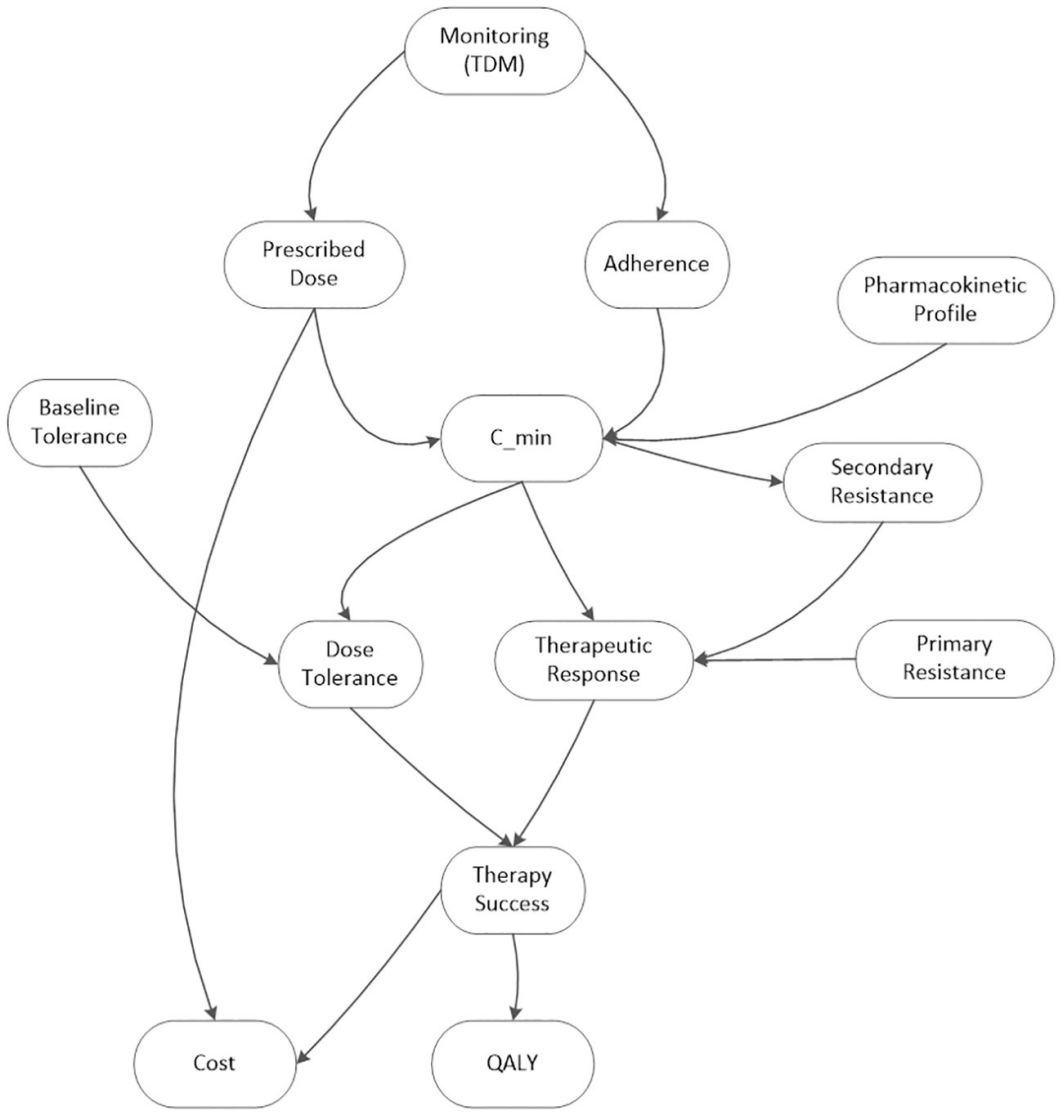
Influence diagram. The diagram shows the relationships between variables in the model. Cost is determined by the prescribed dose and the success of the therapy. The outcome (quality adjusted life years (QALY)) is also determined by therapeutic success. Therapy is successful if the patient tolerates the drug and if there is a therapeutic response. Therapeutic response (cytogenetic or molecular response) fails if there is resistance or if the trough concentration (C_IM_) is inadequate. The trough concentration depends upon the prescribed dose, adherence, and the patient’s pharmacokinetic profile. Dose tolerance depends on the patient’s baseline tolerance and the trough concentration. Therapeutic drug monitoring (TDM) may affect adherence and the prescribed dose. TDM increases adherence. Adherence ensures an adequate trough concentration which, in turn, increases the probability of a therapeutic response. A successful therapeutic response increases the chance that a patient can remain on low-cost imatinib therapy. Thus, adherence lowers costs and increases QALYs.

We assumed that all patients begin treatment with generic IM and are switched to a 2GTKI if IM therapy fails (Fig 1). In our model, the impact of TDM is mediated through its impact on clinical decisions. Our model also factored the two relationships—the effect of TDM on the plasma concentration of IM and the effect of the plasma concentration on therapeutic success of IM—in, and test the sensitivity of the conclusion against the changes in those factors.[20] (Fig 2).

The target population of this study was 55-year-old patients with newly diagnosed CML, which was determined based on the previous population-based effectiveness study.[21] Newly diagnosed patients evolve through a sequence of states (Fig 1). Patients move from state to state in a probabilistic manner that was modeled as a Markov process. All patients start on a standard dose of IM (400 mg/day) as initial therapy and remain on the standard dose while they are responsive. Patients who fail to achieve a complete cytogenic response receive an increased dose of IM (600mg) or switch to a 2GTKI. The dose of IM was decreased to 300 mg when patients developed toxicity or intolerance to the standard 400 mg starting dose. Information from TDM helps physicians decide whether to adjust the IM dose or switch to a 2GTKI.[22, 23] We assumed that patients who had an insufficient response or undue toxicity to 2GTKI were switched to ponatinib, a potent 3^rd^ generation TKI (3GTKI). We assumed that all patients on TKI therapy (IM, 2GTKI or 3GTKI) were in chronic phase (CP). Patients who lose their response to or do not respond to all TKI options may progress (accelerated phase [AP] or blast phase [BP]), and receive a chemotherapy alongside with the 3GTKI until they receive hematopoietic stem-cell transplant (HSCT). The model progresses on a three-month cycle that corresponds to the recommended interval for molecular monitoring.

#### Impact of TDM on response to IM therapy

IM therapy can fail when plasma concentrations of IM are too low. Subtherapeutic concentrations can be caused by poor adherence or by variable pharmacokinetics of IM. TDM can mitigate both of these causes. As mentioned above, regular TDM can encourage adherence, overcome pharmacokinetic variability, and increase the trough level plasma concentration of IM (*C*_*IM*_, the lowest concentration before the next dose is administered). As a result, TDM can assure that IM concentrations are sufficient to promote and maintain a response.

Patients can fail to obtain a response due to sub-therapeutic dosing or due to resistance. Without TDM, physicians are unable to distinguish between these alternatives and would often switch patients to a 2GTKI when they fail to respond to IM therapy. With TDM, physicians can identify patients with sub-therapeutic concentrations and consider adjusting the dose of IM rather than switching to a 2GTKI. Overall, TDM helps patients with inadequate plasma concentrations, either due to poor adherence or to pharmacokinetic variables, obtain and maintain a response to IM and remain on IM rather than switch to a costlier 2GTKI.

### Inputs to simulation model

#### Trough concentration

*C*_*IM*_ depends on many factors including the prescribed dose, adherence, metabolism, and other unknown factors. As a result, there is considerable variation in *C*_*IM*_ even among patients who receive the same dose. We developed a model to predict *C*_*IM*_ based on the prescribed dose and adherence. The model also allows for heterogeneity in the relationship across a population of patients.

Patients are given a prescribed dose, *D*_*P*_, of IM; however, *C*_*IM*_ depends on the actual dose, *D*_*A*_, taken by the patient. *C*_*IM*_ is assumed to be linearly related to the actual dose:

$$C_{IM}={k_{1}D}_{A}={k_{1}D}_{P}A+e$$ (1)

where *k*_1_ = 3.12 is a constant, *A* is the adherence rate and *e* is an error term. We assumed that *e* is explained by *k*_2_ = 0.46, a standard error term for *k*_1_.[24] The error term allows for heterogeneity in the relationship between dosage and *C*_*IM*_ described above. (The derivations of *k*_1_ and *k*_2_ are provided in S1 Appendix).

#### Failure rate for IM therapy

Response to IM therapy is monitored by hematologic, cytogenetic, and molecular criteria. Patients can fail IM therapy due to primary or secondary resistance due to kinase domain mutations. We assumed that 3% of patients experience primary hematologic failure against IM.[25] Hematologic failure is independent of the IM regimen or adherence and is determined 3 months after the initiation of IM therapy.[25]

We developed a model to predict the complete response rate based on duration of IM therapy and *C*_*IM*_ (the failure rate is one minus the response rate). Data for this were taken from a study that estimated response rates following one, two, and five years of IM therapy at different levels of *C*_*IM*_.[26]

$$R_{IM}=0.3197+9.309∙10^{-5}∙C_{IM}-8.911∙10^{-3}\;t+0.2149∙ln(t)$$ (2)

where *R*_*IM*_ is the response rate (percent) and *t* is duration of therapy in the number of three-month cycles. [The derivation of Eq 2 is presented in S2 Appendix] Given an adherence rate of 86.6% to 100%, the equation predicts that 23.2 to 24.8% of patients will fail to respond to IM therapy by month 18. This is consistent with a review on the rate of IM resistance.[25]

#### Intolerance to IM therapy

Intolerance to IM therapy is dose-dependent and intolerance can often be managed by dose reduction.[23, 27, 28] We used data from previous studies that related dose and intolerance to develop a model relating intolerance to *C*_*IM*_. [27, 28]

$$R_{I}={k_{3}C_{IM}}^{2}-k_{4}C_{IM}$$ (3)

where *R*_*I*_ is the intolerance rate per cycle, *k*_3_ = 4.46 × 10^−7^ and *k*_4_ = 1.17 × 10^−4^. This model is based on the relationship between dosage and *C*_*IM*_ (S3 Appendix) and was developed by comparing the cumulative percentage of patients who developed intolerance over the course of IM therapy.[29, 30]

#### Therapeutic target and patient management

**Fig 3:**
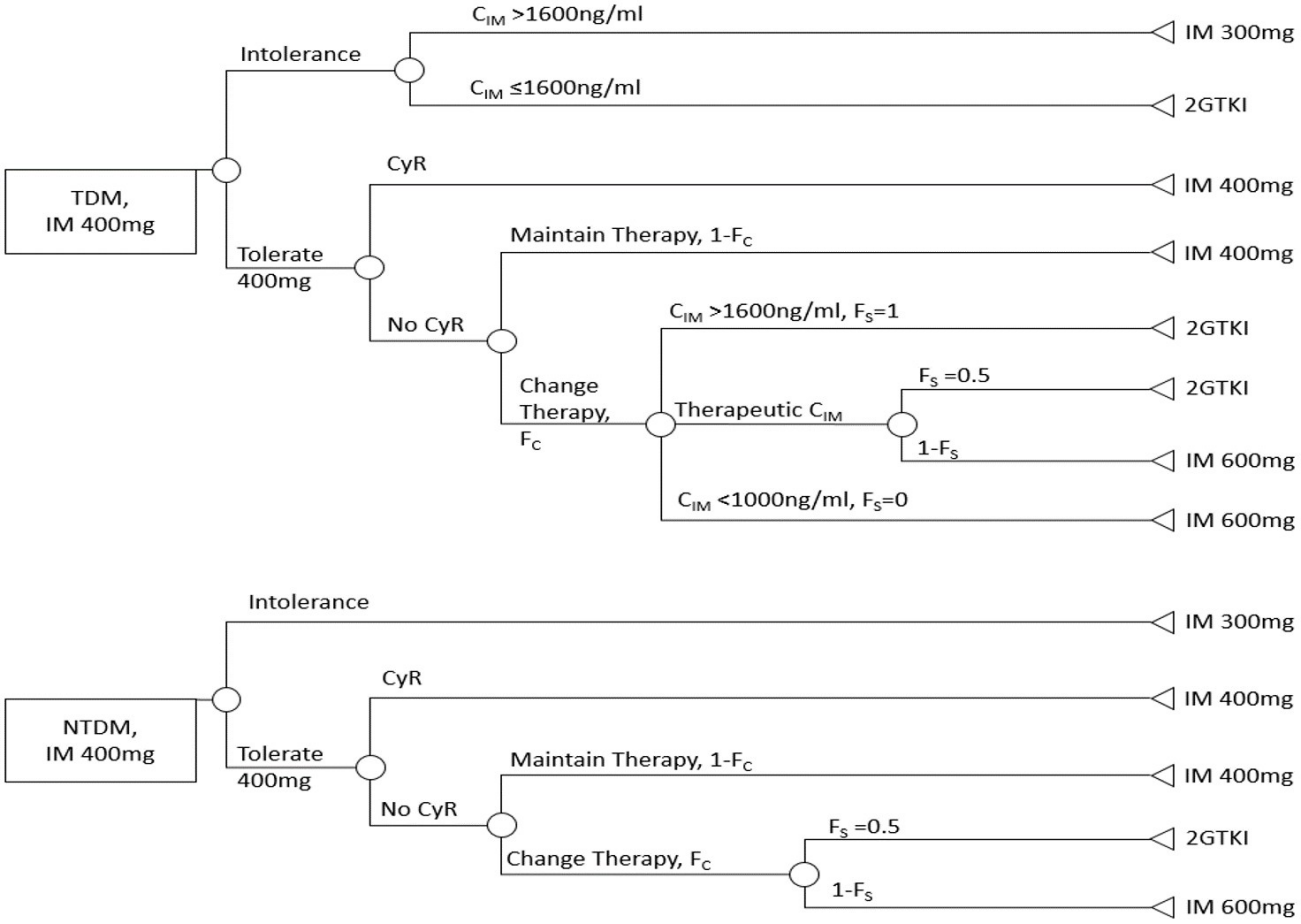
Decision tree for management following treatment failure or intolerance. Abbreviations: CyR = Cytogenetic Response; IM = Imatinib Mesylate; NTDM = No therapeutic drug monitirong; 2GTK I = Second-generation TKI; TDM = therapeutic drug monitoring, TKI = Tyrosine Kinase Inhibitor. *F*_*C*_ = Fraction of patients with change in therapy, varied from 0.25 to 0.75 in sensitivity analysis. *F*_*S*_ = Fraction of patients that are switched to a 2GTKI. *1-F*_*S*_ = Fraction of patients that increase IM dose to 600 mg.

Conventional cytogenetics classifies response to IM into complete (Ph+ 0), partial (Ph+ 1–35%), minor (Ph+ 36–65%), and minimal (Ph+ 66–95%). A fraction (*F*_*C*_) of patients who fail to achieve complete cytogenetic response would receive a treatment change. The TKI option would be selected based on the BCR-ABL1 mutation.[23] Based on expert opinion, we assumed *F*_*C*_ = 0.5 in the base case and varied *F*_*C*_ from 0.25 to 0.75 in sensitivity analysis (Fig 3).

There are two alternative treatment changes: switching to advanced TKIs or increasing IM dose to 600mg (in the absence of IM resistant mutation). The fraction of patients switched to a 2GTKI among all treatment change was *F*_*S*_ in our model. *F*_*S*_ would be informed by *C*_*IM*_ if a patient has been enrolled in TDM. The target concentration for *C*_*IM*_ is between 1000 and 1600 ng/ml.[22, 31–34] IM doses are increased when patients fail to respond and *C*_*IM*_ is below the therapeutic range. On the other hand, TDM patients whose *C*_*IM*_ is above the therapeutic range and fail to respond will switch to 2GTKI. TDM patients whose C_IM_ falls in the therapeutic range and who fail to respond have 50% likelihood of switching to a 2GTKI (*F*_*S*_ = 0.5) or increase to IM 600 mg. In the NTDM arm, physicians have no information regarding *C*_*IM*_ status. The likelihood of treatment switching in those NTDM patients was 0.5. (*F*_*S*_ = 0.5; Fig 3).

#### Failure rate for 2GTKIs and 3GTKI

Patients are placed on a 2GTKI after failing IM therapy. Patients can fail 2GTKI therapy due to resistance or intolerance, and this population switched to ponatinib, a potent 3GTKI. Patients who failed all TKIs progressed to AP. Recent studies for the effectiveness of 2GTKIs estimated the 5-year progression-free survival at between 0.56 and 0.76 in patients who did not show a sufficient molecular response or who were already resistant to IM therapy.[14, 35, 36] Using the midpoint of previous estimates of the 5-year response rate (i.e., 0.66), we estimated that the failure rate, *R*_*F*2_, was 2% per cycle. Of the patients receiving ponatinib, 20% progressed to AP within a year.[9] Based on this, we estimated that the 3-monthly progression rate was 0.054.

#### Rate of HSCT in CML patients

HSCT is generally indicated for all BP patients and AP patients not responding to TKIs, The age-adjusted incidence of CML is 1.6 per 100,000 people which corresponds to about 8,000 incident cases per year.[37] However, only 257 CML patients per year receive HSCTs.[38] Using this number, we calculated that the rate of HSCT per cycle from both post-TKI phases (BP and AP) was 0.33%. The rate of HSCT from each phase was changed between 0% and 3.3% to test the influence of this assumption on the outcome.

#### Adherence rate

Adherence has been shown to be a critical determinant of therapeutic success.[39] We included adherence in the model to investigate the conditions under which TDM would be effective. The expected adherence rate, *A*, was based on a recent observational study, comparing intensive vs. moderate monitoring strategies[40]. The average adherence rate with NTDM (i.e., a moderate level of monitoring) was 86.6%. TDM likely increases adherence; however, the effect of TDM on adherence has not been studied in a CML population. To conservatively estimate the influence of TDM, base-case simulation assumed that TDM does not have influence on the adherence on IM. The possible interaction between TDM and adherence (*R*_*TDM*_) was tested in a sensitivity analysis.

#### Mortality

Patients progress through a series of states (Fig 1). During CP, the mortality rate of CML is similar to the background age-specific mortality rate.[41] We calculated the age-specific mortality during CP using the mortality data obtained from US national vital statistics (NVSS).[42] The three-month survival rate during BP was calculated from the annual survival rate during BP.[11] We estimated the AP mortality by taking the average of the mortality rates during CP and BP. The post-HSCT survival rate (0.988 per three-month cycle), was based on the ten-year post-HSCT survival rate (0.61) from a published study.[43]

#### Costs

We used RedBook to estimate medication costs.[4] The cost of IM was based on the lowest wholesale price of IM. We calculated the cost of 2GTKIs by averaging the cost of three 2GTKIs (dasatinib, nilotinib, bosutinib).[4] Based on this data, the cost of IM was 38% of the cost of 2GTKIs. Patients who fail TKI options were assumed to be on chemotherapy (hydroxyurea, cytarabine, busulfan, cyclophosphamide, vincristine or omacetaxine) for the remainder of their life or until they receive HSCT. We used the wholesale cost of hydroxyurea the low-cost chemotherapy option for CML after resistance TKIs, for the cost of chemotherapy during AP or BP.[4] The cost of Ponatinib from the Redbook was added on top of this chemotherapy cost to estimate the overall expenditure for the advanced CML.[4]

The 2GTKIs will lose patent protection by 2026.[44] Although it is likely that costs will decrease, it is difficult to predict the magnitude and timing of the future changes. For that reason, we did not model any particular cost scenarios for the 2GTKIs. Instead, we performed a sensitivity analysis on the cost of 2GTKIs by varying the cost by ± 25%. The sensitivity analysis may cover some scenarios but it is likely that the cost assumptions regarding 2GTKIs may no longer apply after 2026.

Testing costs (TDM and MM) were obtained from the 2017–2018 (Fiscal Year) Physician Fee Schedule data.[45] The cost of HSCT was obtained from HCUPnet by searching the national statistics for bone marrow transplant.[46] Other costs incurred during CML treatment were extracted from published economic outcome studies.[11, 47, 48] Costs were indexed to July, 2017. Costs were adjusted using the consumer price index (CPI) for medical care components.[49]

#### Quality of life

QALYs were calculated by multiplying the time spent in each state by the corresponding utility score. Utility scores for patients in the CP, AP, and BP states were obtained from published studies.[11, 50–52] Utility for the post-HSCT state depended on graft vs. host disease (GVHD) status. We were unable to find utility weights for the post-HSCT state with GVHD. For that reason, we assumed that the utilities of the post-HSCT state with and without GVHD was equivalent to the utilities of AP and CP, respectively. The utility score for the post-HSCT state was the average of the two (GVHD and non GVHD) weighted by the incidence of GVHD. [52–54] (Table 1) The quality of life in each state includes the impact of medication on the quality of life.

**Table 1:** Inputs for simulation model.

Variable	Input [Distribution for MC]	Reference
Discount Rate, yearly	0.03 [0.01–0.05, Triangular]	
**Cost per 3-month cycle**		
Accelerated Phase Care, Medical	$7,371	[11, 47, 50]
Blast Phase Care, Medical	$7,046	
Chronic Phase Care, Medical	$1,590	
Post-TKI chemotherapy	$440	[4]
Cost of 2GTKI	$37,109 [Triangular, ± 25%]	
Cost of 3GTKI (Ponatinib)	$49,683 [Triangular, ± 25%]	
Generic Imatinib 400mg, daily	$158 [Triangular, ± 25%]	
HSCT, incident cost	$102,654	[46]
Acute Graft vs Host Disease (GVHD), incident cost	$72,913	[11, 48]
Chronic GVHD, incident cost	$11,001	
Post hematopoietic stem cell transplant (HSCT)	$1,590	[11]
Molecular monitoring	$387	[45]
Therapeutic Drug Monitoring	$80 [Triangular, ± 25%]	
**Adherence, C**_**IM**_ **(ng/ml), Response, Intolerance**		
Baseline adherence (NTDM arm), A_NTDM_ (Eq 1)	0.866 [Triangular, 0.654–1]	[68, 71]
*R*_*TDM*_ (Proportion of adherence gap closed by TDM)	0 [0–1 for one-way sensitivity test]	Assumption
C_IM_ (Trough IM concentration [ng/ml], Eq 1)	k_1_ [Normal, SD = k_2_]	[24] S1 Appendix
k_1_and k_2_ for Eq 1	*k*_*1*_ = 3.121, *k*_*2*_ = 0.459	
*R*_*IM*_ (Molecular Response [%], Eq 2)	0.320 + 9.309×10^−5^ × C_IM_ − 8.911×10^−3^ + 0.215 × LN(cycle)	[26] S2 Appendix
*R*_*I*_ (Intolerance [%], Eq 3)	*k*_*3*_× C_IM_2 − *k*_*4*_×C_IM_	[27, 28] S3 Appendix
k_3_ and k_4_ for Eq 3	*k*_*3*_ = 4.46× 10^−7^, *k*_*4*_ = 1.17×10^−4^	
**Clinical Inputs, 3 monthly if not specified**		
Primary hematologic resistance, switching to alternative TKI at 3 months	0.03	[25]
Progression rate, AP to BP	0.1021	[30]
Progression rate, AP or BP to HSCT	0.0033	[72]
Failure rate of 2GTKI therapy, *R*_*F*2_	0.033 [triangular, ±0.02]	[11, 21]
Failure rate of 3GTKI therapy, *R*_*F*2_	0.054 [triangular, ±0.02]	[9]
Incidence of GVHD	0.4	[54]
Survival, BP, multiplied by general survival	0.9461	[11]
Survival, post HSCT, multiplied by general survival	0.9877	[43]
Utility weight*		
Accelerated phase CML	0.79	[11, 50–52]
Blast phase of CML	0.57	
Chronic phase of CML	0.92	
post HSCT, without GVHD	0.98	[11, 52, 53]
post HSCT, with GVHD	0.9 × GHS utility	

* Each utility weight was multiplied by utility of general health status (=0.99)

Abbreviations: C_IM_ = trough concentration of imatinib, CML = chronic myeloid leukemia, C_min_ and C_max_ = decision limits for dose adjustment, MC = Monte-Carlo simulation, NTDM = no therapeutic drug monitoring, TKI = tyrosine kinase inhibitor, 2GTKI = second-generation tyrosine kinase inhibitor, TDM = therapeutic drug monitoring

#### Discount rate

The baseline discount rate was 3%. This rate was applied to both cost and QALYs. The discount rate varied between one and five percent in sensitivity analysis.

#### Base case simulation

We simulated a cohort of 1,000 individuals. The metabolism of IM was heterogeneous as reflected by Eq 1. This scenario used a lifetime horizon and the current cost of IM (38% of the cost of 2GTKI).

#### Sensitivity analysis

Each input parameter was varied within a 95% confidence interval range derived from published literature in a one-way sensitivity test. In some cases, we obtained estimates from clinical experts (sources of input distributions are noted in Table 1). We varied parameters by ±25% from the base case when confidence intervals could not be obtained from the literature or expert opinion.

For probabilistic sensitivity analysis (PSA), we used the results from one-way sensitivity analysis to identify variables that changed the ICER more than $10,000/QALY. We also included the cost of TDM intervention. Distributions for each input variable were obtained from published studies (Table 1). When distributions could not be determined from the literature, a triangular distribution with a ± 25% range was used to model parameter uncertainty. The rates of progression were tested within ±2% point range. The interval was determined based on the 95% confidence limits from previous studies.[21, 28, 55] The parameters were sampled from the respective distributions of the influential variables simultaneously, and simulation was run with those sampled inputs. A thousand simulations for a 1,000-patient cohort was performed.

We also explored some scenarios using a two-way sensitivity test by varying the baseline adherence rate, *A*_*NTDM*_, and the response to TDM, *R*_*TDM*_. The scenarios were designed to determine the benefit of TDM in a best-case situation in which TDM is applied to a hypothetical population with low adherence and a high response rate to TDM.

The price of IM may decrease over time. For that reason, we performed the sensitivity analyses over a wide range of IM prices. Specifically, we performed separate analyses with the cost of IM held at 100%, 75%, 50%, and 25% of the current cost of IM. (The relative cost of IM to 2GTKIs was 38%, 29%, 19%, and 10%).

#### Treatment free remission scenario

In this scenario, treatment was discontinued for patients who received 400 mg IM for at least 3 years and had achieved a deep molecular response for two years or longer.[56] We addressed the influence of TFR on the economic outcome assessment using a separate Markov Status (S4 Appendix). Patients who received 400 mg IM longer than 3 years were eligible to move to TFR.[57, 58] We addressed the influence of TFR on the economic outcome assessment using a separate Markov Status (S4 Appendix). The likelihood of TFR was estimated using published data on the cumulative incidence of TFR as a function of the duration of IM therapy (S4 Appendix).[57] Patients may experience a relapse during TFR. The likelihood to of being IM discontinued for TFR was determined based on the logarithmic regression of the cumulative incidence of TFR on the duration of IM using published data. The likelihood of relapse while patients are on TFR was calculated from the exponential regression assuming 50% of TRF patients relapsed in 2 years from the beginning of the TFR. (S4 Appendix)[56, 59] The relapse rate depends on the length of IM therapy prior to TFR. In the optimal case (e.g., 8+ years on IM therapy), progression to AP from TFR is negligible.[60] However, patients sometimes begin TFR after shorter periods of IM therapy (e.g. three years). Shorter IM therapy is associated with higher relapse rates. In optimal case (e.g., 8+ years on IM therapy), progression to AP from TFR is negligible. However, discontinuation of IM sometimes incurs in patients receiving IM regimen as short as 3 years which is associated with a suboptimal outcomes of TFR.[57–59] To account for the difference in the optimal scenario vs. real-world practice, our model assumed that a 10% of patients who relapsed from TFR will progress to AP directly. At the current price of IM and TDM, the influence of the inclusion of the TRF on cost and QALY were assessed for the three time horizons: 5 years, 10 years and lifetime.

#### Time horizon

Different stakeholders focus on different time horizons. For example, a short-term perspective may be relevant for a health insurer and a lifetime perspective might be relevant from a government perspective. For that reason, we examined the cost effectiveness in five-year increments from diagnosis until death.

## Results

### Base case analysis

**Fig 4:**
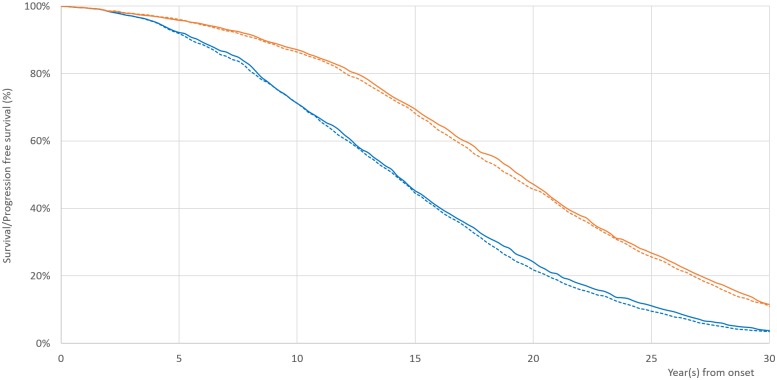
Progression-free survival and survival estimates for TDM vs. NTDM. Progression-free survival (PFS, blue) and survival (orange) estimates. Estimates from TDM on solid lines; estimates from NTDM on dotted lines. Note: Progression-Free Survival: likelihood of staying in CP with either imatinib or second-generation TKI. Abbreviations: NTDM = No therapeutic drug monitoring; TDM = therapeutic drug monitoring.

TDM was associated with an increase in cost and an increase in QALYs. In the TDM arm, the average lifetime cost of treatment was $2,136,677 [95% CI: 2,079,406 2,174,412] and patients accumulated 12.37 [95% CI: 12.07; 12.55] QALYs. In the NTDM arm, the cost of treatment was $2,132,260 [95% CI: 2,091,296; 2,196,606], and patients accumulated 12.23 [95% CI: 11.96; 12.50] QALYs. Thus, on average, patients in the TDM arm experienced a small gain in QALYs (0.15: 95% CI:-0.13;0.28]) for an increase in cost ($4,417[95% CI: -52,582;32,098]). The incremental cost-effectiveness ratio for TDM was $30,450/QALY. The estimated ten-year survival rates for TDM and NTDM were 86.6% and 85.8%, respectively. These survival estimates are consistent with the estimate from a recently published open label multicenter trial (Table 2, Fig 4).[61]

**Table 2:** Results from base case analysis and TFR scenario.

	Result	Time Frame					
		Five year		Ten year		Life Time	
		TDM	NTDM	TDM	NTDM	TDM	NTDM
Base Case Scenario	Total Cumulative Cost ($)	473k	484k	1,045K	1,064K	2,137k	2,132K
	Quality adjusted life years (QALY)	4.23	4.24	7.49	7.47	12.37	12.23
	Overall Survival (%)	95.8	96.1	87.1	86.4	-	-
	Progression-Free Survival (%)	92.2	91.9	87.1	86.4	-	-
	% on Imatinib	38.8388	36.6	13.3	12.7	-	-
	Intolerance to IM, Cumulative	5.1%	4.5%	7.9%	6.9%	8.6%	7.7%
TFR Scenario	Total Cumulative Cost ($)	385K	389K	897K	912K	1,989K	1,987K
	Quality adjusted life years (QALY)	4.12	4.12	7.35	7.35	12.29	12.23

Cost and QALY are discounted by the annual rate of 3%.

Abbreviations. TDM, therapeutic drug monitoring. TFR, treatment-free remission, NTDM, No therapeutic drug monitoring.

### Sensitivity analysis

**Fig 5:**
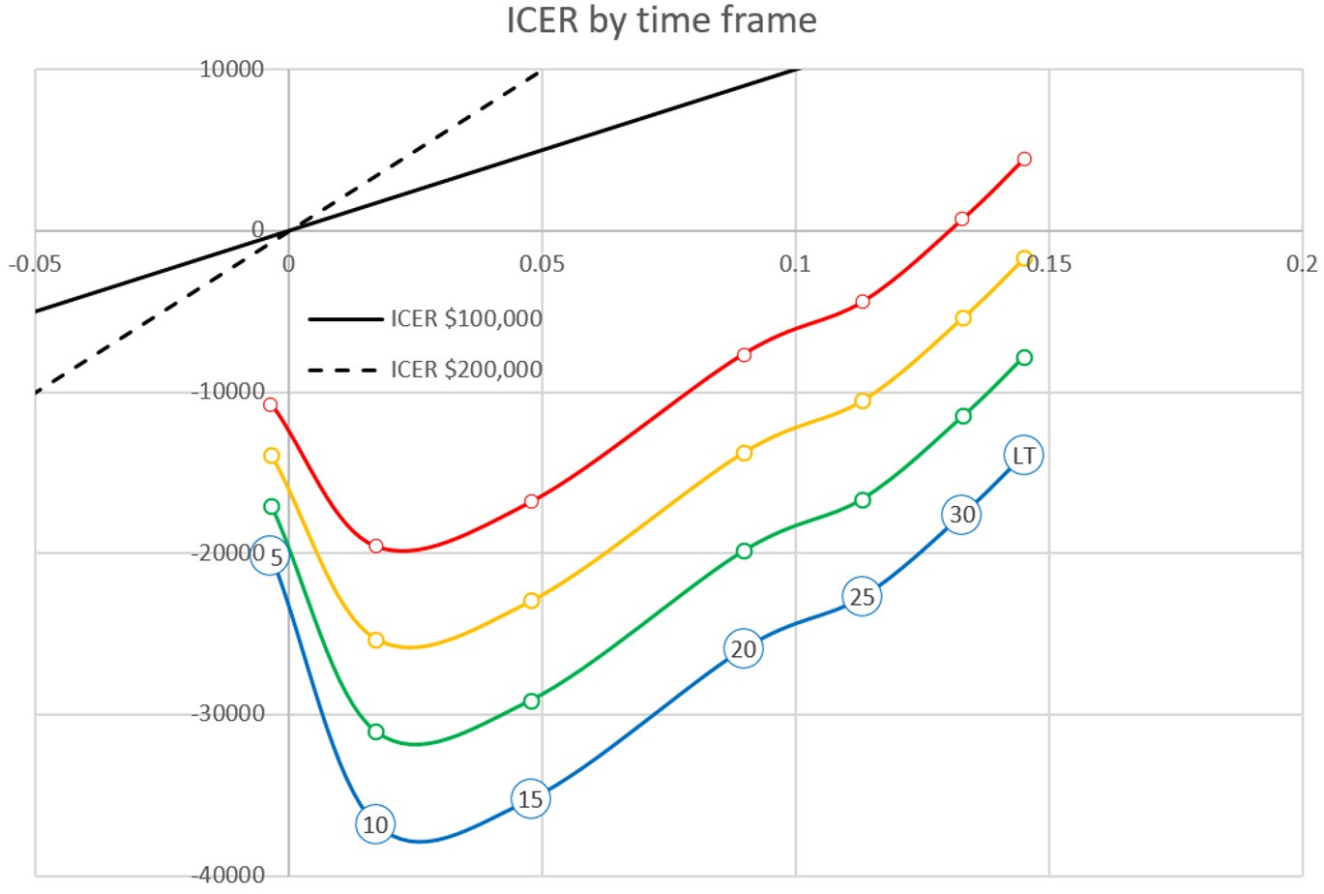
Influence of timeframe, the cost of IM, cost of BP medication on the incremental cost-effectiveness ratio. X-axis, Incremental Quality-Adjusted Life Years (QALY) gained; Y-axis, Incremental Cost in USD. Red: The cost of IM is 38% of the cost of second-generation tyrosine kinase inhibitor (2GTKI)–Current. Orange: The cost of IM is 29% of the cost of 2GTKI, 75% of current IM cost. Green: The cost of IM is 19% of the cost of 2GTKI, 50% of current IM cost. Blue: The cost of IM is 10% of the cost of 2GTKI, 25% of current IM cost. Numbers in circle markers: Model time frame (years); Abbreviations: ICER = Incremental Cost-Effectiveness Ratio ($US per QALY).

The cost effectiveness was sensitive to the time horizon of the analysis. TDM reduced total costs over intermediate horizons (five to ten years) but increased costs over horizons longer than ten years (Fig 5). TDM reduced costs by $19,548 and gained 0.02 QALYs per patient over a ten-year timeframe.

A scenario analysis showed the decrease in the cost of generic IM was associated with additional savings in the cost of the TDM strategy. For example, the ten-year cost savings were $25,299, $31,050 and $36,802, when the cost of IM was reduced by 25%, 50% or 75%. TDM reduced costs over a lifetime horizon if the cost of IM was decreased to 75% of the current cost. (Fig 5) Changing the rate of HSCT from AP or BP between 0% and 3.3% varied the lifetime ICER between $327/QALY and $37,580/QALY, which favors TDM at a willingness to pay (WTP) threshold of $50,000/QALY.

**Fig 6:**
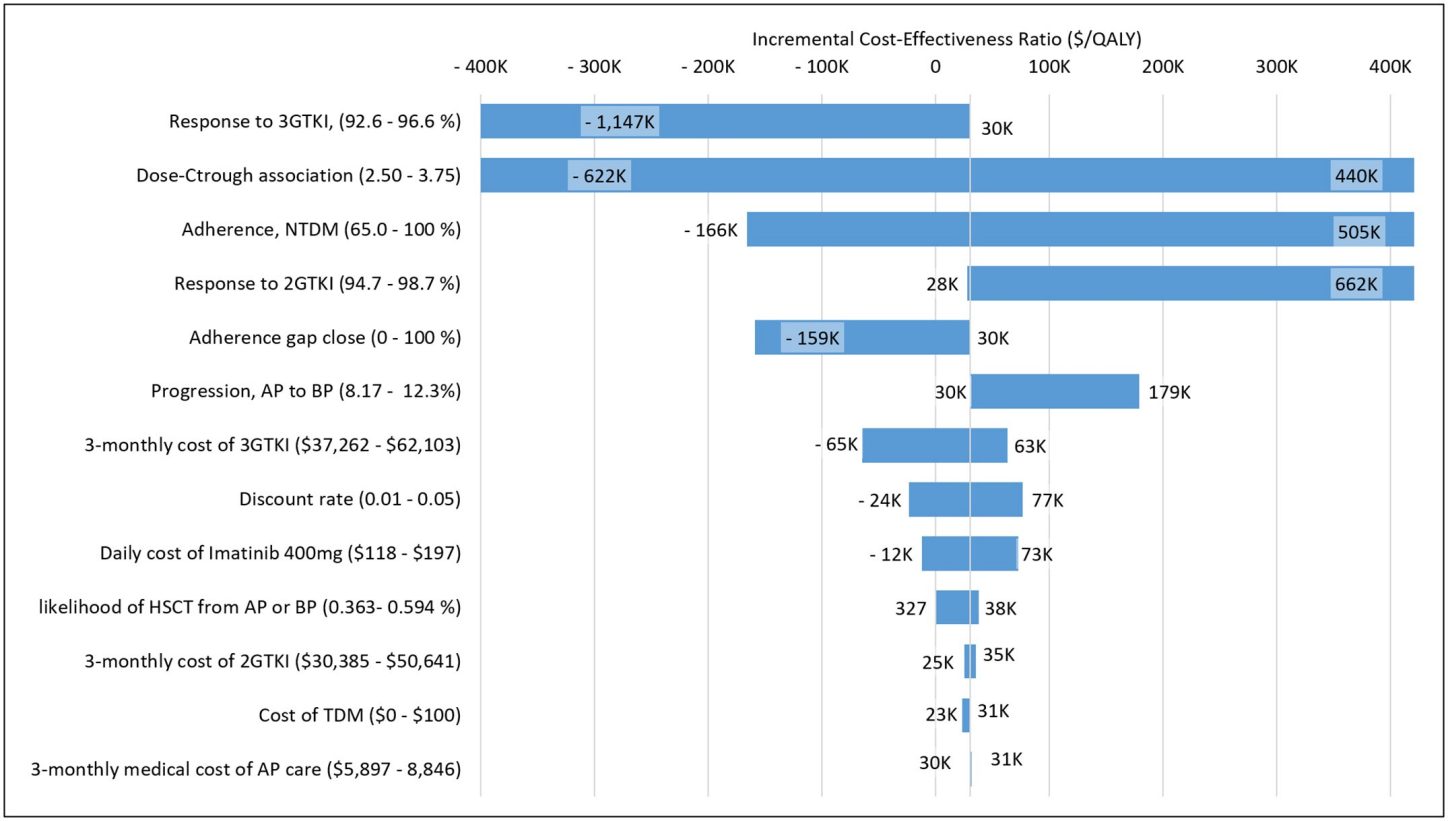
One-way sensitivity analysis. The graph shows the change in the incremental cost effectiveness ratio (ICER) due to variation of a single input. Inputs are listed on the left along with the range over which the input was varied. Abbreviation**s**. A_NTDM_ = adherence in the absence of TDM; AP = accelerated phase; BP = blast phase; C_IM_, = trough concentration of imatinib mesylate; IM = imatinib mesylate; NTDM = No therapeutic drug monitoring; TDM = therapeutic drug monitoring.

Six the most influential variables from the one-way sensitivity analysis were response to 3GTKI, association between the IM dose and *C*_*IM*_ (*k*_*1*_), baseline adherence (*A*) in NTDM, response to 2GTKI therapy (*R*_*F*2_), influence of TDM on adherence (*R*_*TDM*_), and the rate of progression from AP to BP. The ICER was also sensitive to the relationship between dose and *C*_*IM*_ (*k*_*1*_,Eq 1) (Fig 6). Among these variables, the ICER exceeded a WTP of $100,000 when *k*_*1*_ *was* greater than 3.25 or the baseline adherence greater than 0.90. The one way sensitivity test result of the other four variables crosses x-and y-axis of the ICER plane, which resulted in an ICER greater than $100,000 when both incremental cost and incremental effectiveness were negative. However, those ICERs were never greater than the WTP threshold line within the suggested range for the sensitivity analysis.

The ICER was relatively insensitive to changes in the costs of 2GTKIs. The ICER varied from $35,431/QALY to $25,465 when the average cost of 2GTKIs changed from $30,385 to $50,641.

**Fig 7:**
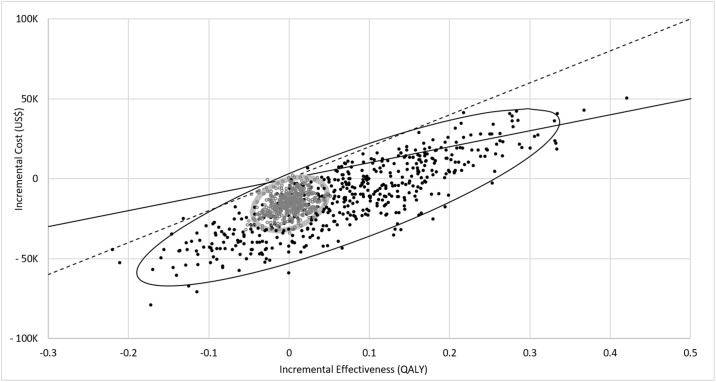
Incremental cost-effectiveness ratio estimates from a monte carlo simulation conditional on the current cost of IM from RedBook^®^. Gray Dots: Results from a 500-cohort simulation for a 10-year timeframe. Black Dots: Results from a 500-cohort simulation for a lifetime. Solid line: Willingness to pay (WTP) threshold of $100,000/QALY gained. Simulations below this line represent the acceptance of TDM at WTP threshold of $100,000/QALY gained; Dotted line: Willingness to pay threshold of $50,000/QALY gained. Simulations below this line represent the acceptance of TDM at WTP threshold of $50,000/QALY gained. Each cohort simulation represents the mean incremental cost effectiveness of 1,000 microsimulations.

**Fig 8:**
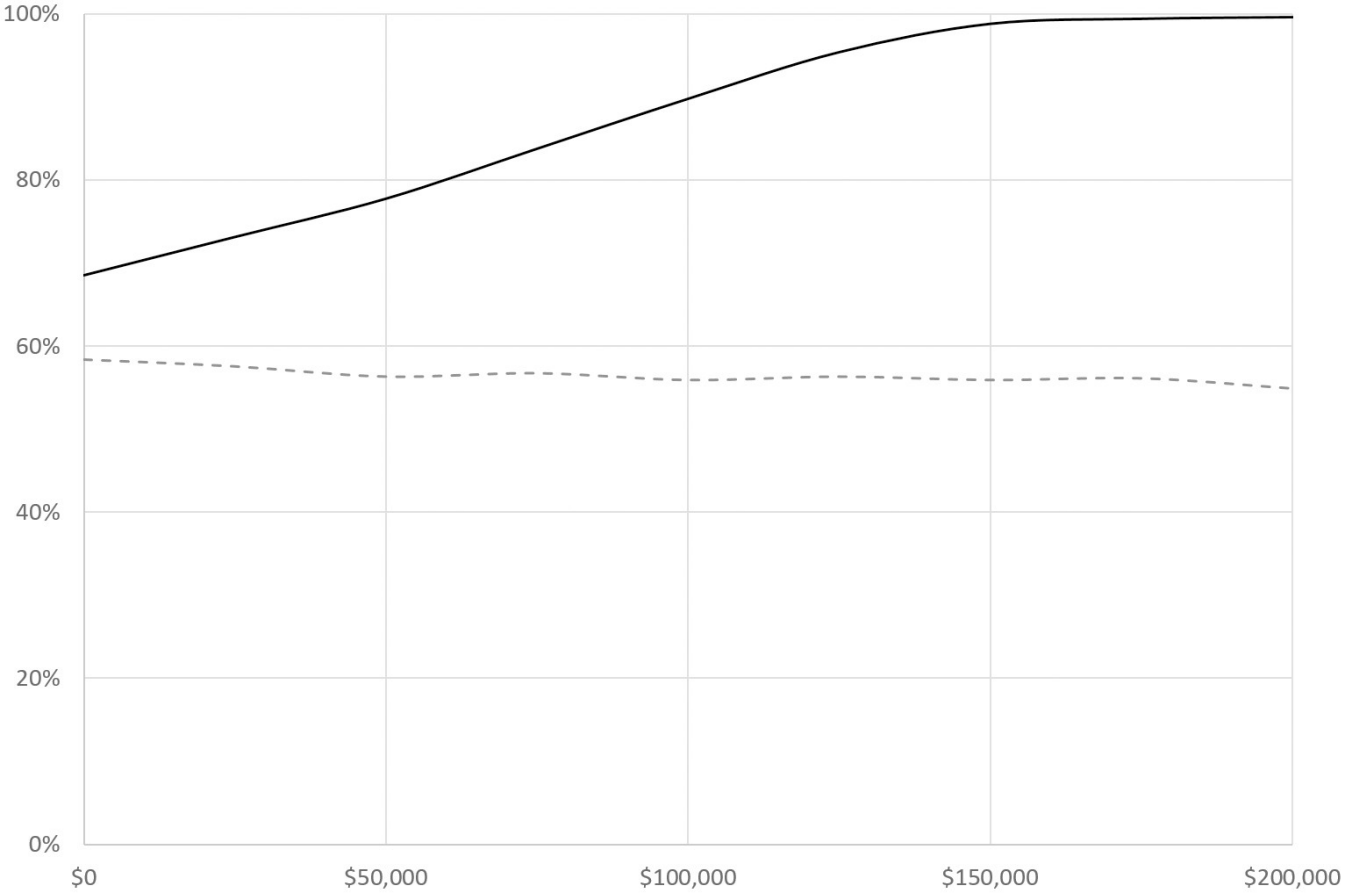
Cost-effectiveness acceptability curve from a 1,000 cohort simulation. The acceptability curve represents the likely of TDM being accepted depending on the price of imatinib at varying levels of willingness-to-pay threshold. Solid line: Likelihood of TDM being accepted, decision based on the lifetime incremental cost effectiveness ratio. Dotted line: Likelihood of TDM being accepted, decision based on the ten-year incremental cost effectiveness ratio.

Probabilistic sensitivity analysis showed that TDM was more cost effective than NTDM in 89.77% of the simulations based on a lifetime horizon and a WTP of $100,000/QALY, a common threshold (Figs 7 and 8). The acceptability of TDM increased with the increase in willingness to pay (WTP) threshold, and reached 99% at the WTP of $175,000 per QALY gained. TDM saved cost in 58.31% and 98.59% of the simulation when the time horizon was to five and ten years, respectively (Figs 7 and 8).

### Treatment free remission scenario

Being attributable to the discontinuation of TKI, treatment cost decreased in both TDM and NTDM arms without a significant gain or loss of QALY when the TFR scenario was simulated. In the TDM arm, the average lifetime cost was $1,989,450 and patients accumulated 12.29 QALYs. In the NTDM arm, the cost of treatment was $1,987,190, and patients accumulated 12.23 QALYs. Thus, accounting for the discontinuation of IM for TFR, patients in the TDM arm experienced a small gain in QALYs (0.06) and a decrease in cost ($2,257). TDM was a preferred option from the 5-year and 10-year assessment for the TFR scenario, too. (Table 1).

## Discussion

We evaluated the cost effectiveness of TDM in patients treated with IM for CML. Our study demonstrated that clinical decisions informed by the plasma concentration of IM from a TDM services increases costs and provides a small gain in QALY relative to NTDM when analyzed over a lifetime horizon. The incremental cost-effectiveness ratio was lower than $100,000/QALY, a conventional WTP threshold. Therapeutic decision informed by the trough level of imatinib likely increases the duration of IM therapy and reduces the need to switch to more expensive therapies (2GTKIs or 3GTKI). If TDM could improve the level of adherence, as tested in the one-way sensitivity analysis, the incremental cost per QALY gain from the TDM arm would become less. TDM was associated with an increase in lifetime costs because patients in the TDM arm lived longer and accumulated more health care costs. TDM reduced costs over shorter time frames (five or ten years). Across the three different time-horizons including five years, ten years and lifetime, the likelihood of TDM being accepted was greater than 95% with a WTP threshold of $200,000/QALY, an upper cost-effectiveness limit based on the World Health Organization’s suggestion (i.e., 3 times of the GDP per capita), meaning that there TDM is likely cost-effective over a lifetime horizon and, based on our simulations, there is relatively little uncertainty associated with this conclusion.

Our estimate of the ten-year survival rate from the NTDM (86%) was slightly higher than the point estimate from a recent open-label multicenter trial but fell within the 95% confidence interval of that estimate.[61] Considering this multi-center trial started in early 2000, our study reflects the improved survival associated with advanced treatment options. Our estimate of the three-year progression free survival rate (96.5% for TDM, 96.7% for NTDM) was higher than the estimate (mean 94%, 95% CI, 92–95%) from a randomized trial that allowed for a tolerability-adjusted IM dose increase for CML patients.[55] This outcome was also reliable in reference to the improved CML care over the last decade. The first year progression-free survival against TKI was improved from 95–98% in early 2000s to 100% in a recent study.[21, 62–64] The intolerance rate was within the range (7% to 14%) reported in previous studies.[29, 30] The calculated five-year or ten-year rate of discontinuation from our study was about 30 percent higher than the results from some previous randomized trials.[61, 65] Nevertheless, the estimates from our model were very similar to the results from observational studies.[66, 67] In general, the results of our study are consistent with other studies.

The lifetime cost estimate from our model is substantially higher than the 2014 estimate for the cost of treatment in CML patients of IM or 2GTKI followed by another 2GTKI and chemotherapy.[11] The difference is predominantly attributable to the treatment options for post-2GTKI stages. For example, the patients from the Rochau’s study moved to chemotherapy once they failed a 2^nd^-line option. However, in our study, a large proportion of patients who fail 2GTKI are still in CP and they have an opportunity to receive a 3GTKI before their CML progress. Further, recent clinical practice put patients on a TKI in combination with chemotherapy even after they have progressed to AP or BP. The difference in cost estimates was also due, in part, to inflation in the cost of medical care (9%), and the increase in the price of 2GTKIs (31 to 58%) between 2014 and 2017.[4, 49] Another study, conducted by Padula et al., estimated the five-year cost of CML care using IM.[10] This study assumed that the price of IM will decrease significantly in the year after IM becomes generic and modeled the impact of various levels of price decrease (up to 90%). They estimated that the five-year cost would be $260K when the cost of generic IM is 25% of the listed price.[10] Thus Padula’s result was reproduced from our study when our simulation combined two scenarios, switching from 2GTKI to AP chemotherapy and a large drop in IM price. The five-year cost estimates under these assumptions were $269K for TDM and $279K for NTDM. Although our modeling approach differs from previous modeling studies (mechanistic vs. empiric), our model produces similar and/or reliable outcomes. The cost differences can be explained by the changes in CML care over the past decade.

Our study showed that universal testing is cost effective. However, it is likely that targeted strategies would be more cost-effective or cost-saving. Average adherence is high (86.6%),[68] and TDM will not be cost effective in an adherent subgroup because there is little or no room for improvement. TDM has more potential to be cost effective in subgroups with low adherence. Thus, it would be useful to know the impact of TDM on these groups and, if they respond to TDM, to devise strategies to focus on these groups. For example, one might limit TDM to patients who show poor response or reduce the frequency of TDM testing among responders. The results of our two-way sensitivity analysis suggest that either of these strategies would be likely to make TDM more cost effective. Also, the cost of TDM could be reduced if it were only employed universally during an initial “dose finding” period.

Although we found that TDM is cost effective, the impact of TDM was relatively modest. On average, TDM was associated with a gain of 0.15 (95% CI:-0.13; 0.28) QALYs and a cost increase of $4,417 (95% CI: -52,582; 32,097). In addition, there is considerable uncertainty in the estimates: the confidence intervals for QALYs and cost are large relative to the average.

Our model is based on the premise that TDM would allow for more effective management of patients and, in turn, would result in lower costs and better outcomes. Part of the cost savings is based on the assumption that imatinib is less expensive than the alternatives. This is true at present because imatinib is available as a generic drug while dasatinib, bosutinib and nilotinib are still under patent. These 2GTKIs will lose patent protection over the next four to seven years (bosutinib: 2026; dasatinib: 2025; nilotinib 2023; ponatinib 2026).[44] It is likely that the cost of the 2GTKIs will decrease relative to imatinib once they go off patent. Our sensitivity analysis showed that the ICER was relatively insensitive to decreases of up to 25% in the cost of 2GTKIs; however, it is very difficult to predict the timing and magnitude of the change. Thus, our analysis is limited by the fact that the underlying assumptions regarding the cost of 2GTKIs will most likely need to be revised in 2026. TDM appears to be cost effective given the current pricing of 2GTKIs.

We used a simple average cost of the 2GTKIs for the cost of a 2GTKI. In principle, a market-share weighted average would be better; however, we were unable to obtain market share data. It turns out that the prices of the 2GTKIs for in a fairly narrow range. The maximum deviation of the price of an individual 2GTKI from the mean price was 10% so it is unlikely that the difference between the simple average and weighted average would be practically significant. In addition, we performed sensitivity analysis in which the price of 2GTKI varied 25%.

Our approach differs from previous studies because we used a mechanistic model based on the underlying pharmacokinetics and pathophysiology. In our model, TDM has two impacts: increasing adherence and improving the investigation of relapse or therapeutic failure. We modeled the effect of adherence on the trough concentration of IM and, using data from published studies, related the trough concentration to response. Similarly, we modeled the impact of TDM on clinical decisions. Our approach allowed us to explore a range of scenarios that would not be possible in previous models. [69, 70]

Our study has several limitations. The inputs to our model were based on costs and practices in the United States. Therefore, our results are limited to this context. Therapeutic drug monitoring may be more costly or unavailable in other contexts. Also, practice may differ in other contexts. For example, we assumed that ponatinib would be used as a third line TKI which is not the practice in Europe.

Our study also has several strengths. We explored the cost effectiveness of TDM over a wide range of prices for IM and over several time horizons. Also, we developed a mechanistic model that relates monitoring to dosing, tolerance, and adherence. Our objective was to predict whether TDM is likely to be cost effective. Our results suggest that it could be; however, this needs to be shown empirically. Our results suggest that such a study is likely to be worthwhile.

## Conclusions

Universal TDM reduces the cost of CML treatment over a five- to ten-year horizon (insurance payer’s perspective). Universal TDM increases the overall cost but is acceptable (86.2862% at WTP of $100,000 per QALY) when applied to a lifetime horizon.

## Supporting information

S1 Appendix Dose—C_IM_ association. (DOCX)

S2 Appendix C_trough_—Response association (data from larson et al.). (DOCX)

S3 Appendix C_IM_—Intolerance association data. (DOCX)

S4 Appendix State transition diagram for treatment free remission scenario. (DOCX)

S1 Data (TREX)
