# Supplementary material for: Cost effectiveness of therapeutic drug monitoring for imatinib administration in chronic myeloid leukemia
Source: PLoS One. 2019 Dec 23;14(12):e0226552. doi: 10.1371/journal.pone.0226552 (PMC6927594; doi:10.1371/journal.pone.0226552)
Supplement: S1 Appendix — (DOCX) [file pone.0226552.s001.docx]

**APPENDIX I.**

**Dose – C_IM_ Association**

| Dose | Mean C_IM_ (ng/ml) | 95% CI, Lower Limit | 95% CI, Upper Limit |
| --- | --- | --- | --- |
| 100 | 210 | 5 | 415 |
| 200 | 715 | 548 | 882 |
| 300 | 940 | 741 | 1139 |
| 400 | 1226 | 836 | 1616 |

Dose – C_IM_ Estimate from a linear regression model using the Yoshida data

Forest Plot: Mean and 95% confidence intervals of C_IM_ at each dose, Data from Yoshida et al.

Black dotted line: Mean C_IM_ estimate from linear regression. $C_{IM}=k_{1}\times dose \left[ mg \right],$

Gray dotted line: 95% confidence interval estimate from linear regression

$$\therefore C_{IM}=f\left( dose \left[ mg \right] \right)= k_{1}\times dose \left[ mg \right]$$

$$where k_{1} \sim N [3.12, \left( k_{2})^{2} \right], k_{2}=0.46$$

**Source of data.**

- Yoshida, C., et al., Adherence to the standard dose of imatinib, rather than dose adjustment based on its plasma concentration, is critical to achieve a deep molecular response in patients with chronic myeloid leukemia. Int J Hematol, 2011. 93(5): p. 618-23.
