# Supplementary material for: Cost effectiveness of therapeutic drug monitoring for imatinib administration in chronic myeloid leukemia
Source: PLoS One. 2019 Dec 23;14(12):e0226552. doi: 10.1371/journal.pone.0226552 (PMC6927594; doi:10.1371/journal.pone.0226552)
Supplement: S2 Appendix — (DOCX) [file pone.0226552.s002.docx]

**APPENDIX II.**

**C_trough_ – Response Association** (Data from Larson et al.)

| Steady State C_IM_ (ng/ml) | Response Rate | | |
| --- | --- | --- | --- |
|  | Year 1 | Year 2 | Year 5 |
| 490 | 0.59 | 0.73 | 0.83 |
| 889 | 0.71 | 0.80 | 0.88 |
| 1661 | 0.73 | 0.84 | 0.93 |

**Response rate over time (estimate from SAS version 9.4)**

$$Response=f\left( C_{IM}, cycle \right)$$

$$= 0.3197+9.309\cdot{10}^{-5}\cdot C_{troughIM}-8.911\cdot{10}^{-3}\cdot cycle+0.2149\cdot ln(cycle)$$

**Response rate estimate from a natural log function**

Circular markers: data from Yoshida et al.

Lines: estimates from the natural log function

Blue: Steady state C_IM_ of 490 g/ml

Orange: Steady state C_IM_ of 889 g/ml

Green: Steady state C_IM_ of1661 g/ml

**Source of data.**

- Larson, R.A., et al., Imatinib pharmacokinetics and its correlation with response and safety in chronic-phase chronic myeloid leukemia: a subanalysis of the IRIS study. Blood, 2008. 111(8): p. 4022-8.
