# Supplementary material for: Cost effectiveness of therapeutic drug monitoring for imatinib administration in chronic myeloid leukemia
Source: PLoS One. 2019 Dec 23;14(12):e0226552. doi: 10.1371/journal.pone.0226552 (PMC6927594; doi:10.1371/journal.pone.0226552)
Supplement: S3 Appendix — (DOCX) [file pone.0226552.s003.docx]

**APPENDIX III.**

**C_IM_ – Intolerance Association data**

| Dose | C_IM_ (ng/ml) estimate from APPENDIX II | 12-month Intolerance requiring dose reduction or IM discontinuation | Per cycle  rate |  |
| --- | --- | --- | --- | --- |
| 400mg | 1249 | 0.000 | 0.0000 | Talpaz et al.  Blood 2002 |
| 600mg | 1872 | 0.050 | 0.0126 |  |
| 400mg | 1249 | 0.038 | 0.00950 | Cortes et al.  JCO 2010 |
| 800mg | 2497 | 0.094 | 0.0235 |  |

**C_IM_ – Intolerance regression**

Incidence rate (%) = 4.46 × 10^-7^ × C_IM_^2^ – 1.17 × 10^-4^ × C_IM_, R^2^ = 0.84

**Source of data.**

- Talpaz, M., et al., Imatinib induces durable hematologic and cytogenetic responses in patients with accelerated phase chronic myeloid leukemia: results of a phase 2 study. Blood, 2002. 99(6): p. 1928-37.
- Cortes, J.E., et al., Final 5-Year Study Results of DASISION: The Dasatinib Versus Imatinib Study in Treatment-Naive Chronic Myeloid Leukemia Patients Trial. J Clin Oncol, 2016. 34(20): p. 2333-40.
