# Supplementary material for: Cost effectiveness of therapeutic drug monitoring for imatinib administration in chronic myeloid leukemia
Source: PLoS One. 2019 Dec 23;14(12):e0226552. doi: 10.1371/journal.pone.0226552 (PMC6927594; doi:10.1371/journal.pone.0226552)
Supplement: S4 Appendix — (DOCX) [file pone.0226552.s004.docx]

**APPENDIX IV.**

**State Transition Diagram for Treatment Free Remission Scenario**

Patients who received the initial dose imatinib for 3 years or longer are eligible to transit to the treatment-free remission status. Rate of remission and relapse is calculated based on the published data

1.2

Dose

Escalation

1.3

Dose

De-escalation

2.

Secondary

TKI,

Chronic Phase

3.1

Accelerate

Phase

3.2

Blast

Phase

3.3

Post

HSCT

1. Imatinib,

Chronic Phase

3. Post TKI

4.

Death

1.1

Initial

Dose

[Scenario]

Treatment-Free Remission

Remission

Relapse

**Cumulative Incidence of Treatment Free Remission (TFR)**

Cumulative Incidence of TFR = 0.4328 · ln(cycle) – 1.155, R^2^ = 0.9281

Calculation applied to patients who were on the imatinib 400mg longer than 3 years (i.e., 12 cycles)

Source of data:

- Shanmuganathan N., et al., Cumulative Incidence of Treatment-Free Remission (TFR) for Patients with Chronic Myeloid Leukemia (CML): The Adelaide Experience. Blood, 2017. 130: 1621.

| Duration of TKI therapy | Cumulative Incidence of  TKI cessation attempted |
| --- | --- |
| 5 years | 4% |
| 10 years | 40% |
| 10 < years: considered up to 15 years for our calculation | 68% |

**Rate of relapse from TFR**

Approximate remission free at 2 years (8 cycles) = 50%.

Rate of relapse per cycle = 1 - EXP(ln(0.5)/8) = 0.083

Source of data and assumption:

- Laneuville P., When to Stop Tyrosine Kinase Inhibitors for the Treatment of Chronic Myeloid Leukemia. Curr Treat Options Oncol. 2018;19(3): 15.
- Ross M et al., Safety and efficacy of imatinib cessation for CML patients with stable undetectable minimal residual disease: results from the TWISTER study. Blood. 2013;122(4):515-22
